# Home-Combo: an online home-based exercise intervention for women with breast cancer undergoing neoadjuvant chemotherapy: study protocol for a 2-arm pragmatic randomized controlled trial

**DOI:** 10.3389/fonc.2025.1682839

**Published:** 2025-12-12

**Authors:** Pedro G.F. Ramos, Eliana V. Carraça, Gabriela Valadas, Rui M. Batalau, António L. Palmeira, Marlene N. Silva, Patrícia C. Viegas, Jorge G. Oliveira, Sara S. Pereira, António Videira-Silva, Isabel F. Santos, Sofia Franco, Diana Gomes, Daniela Teixeira, Nathalie G. Camilo, Inês Nobre, Nuno Dias, João Pais, Diana Koshman, Pedro B. Júdice

**Affiliations:** 1 Centro de Investigação em Desporto, Educação Física, Exercício e Saúde (CIDEFES), Universidade Lusófona Centro de Lisboa, Lisbon, Portugal; 2 Formação, Inovação e Intervenção em Desporto (CIFI2D), Porto, Portugal; 3 Local Health Unit Algarve, Faro, Portugal; 4 Centro de Investigação em Desporto, Educação Física, Exercício e Saúde (CIDEFES), Instituto Superior Manuel Teixeira Gomes, Portimão, Portugal; 5 Center for Other Worlds (COW), Universidade Lusófona Centro de Lisboa, Lisbon, Portugal; 6 Digital Human-Environment Interaction Lab (HEI-Lab), Universidade Lusófona Centro de Lisboa, Lisbon, Portugal; 7 Centro Interdisciplinar de Performance Humana (CIPER), Faculdade de Motricidade Humana, Cruz Quebrada-Dafundo, Portugal; 8 Centro de Investigação em Desporto, Educação Física, Exercício e Saúde (CIDEFES), Lusófona University of Humanities and Technology, Lisboa, Portugal

**Keywords:** breast cancer, home-based exercise, neoadjuvant chemotherapy, relative dose intensity, tumor regression, pathological complete response

## Abstract

**Background:**

Chemotherapy carry side effects and potentially result in dose decreases or treatment delays of women with breast cancer (BC). Relative dose intensities of chemotherapy <85% are associated with a worse disease prognosis and lower treatment efficacy. Exercise may modulate treatment response through its effects on tumor microenvironment. Home-based exercise is a convenient strategy, proven to be effective and beneficial for women with a BC diagnosis under treatment. Recent evidence suggests that exercise may contribute to tumor regression neoadjuvant treatment. Studies investigating the effects of exercise interventions on chemotherapy completion rates are needed. No study appears to have analyzed the effects of a home-based exercise interventions on tumor regression in women with BC. This paper describes the protocol of a 2-arm pragmatic randomized controlled trial, targeting women with a BC undergoing neoadjuvant chemotherapy.

**Methods:**

A 2-arm randomized controlled trial implemented in a real-world exercise setting to compare an online structured and supervised group exercise intervention, with an active control group, during the neoadjuvant chemotherapy phase. Ninety-eight participants with a BC diagnosis stage I-III, scheduled to have neoadjuvant chemotherapy will be enrolled continuously for one year. The primary outcomes of this study will be chemotherapy completion rate and tumor regression. Secondary outcomes will include, body composition, functional performance, and self-reported physical activity levels and general and BC-specific quality of life. Outcome measures will be obtained at baseline, mid-treatment, post-intervention, and 3-month follow-up.

**Discussion:**

Home-based exercise training programs are safe for women with BC during the treatment. Studies conducting structured and supervised home-based exercise programs in women with BC during the treatment are still scarce. Since this study involves participants performing neo-adjuvant chemotherapy, it will allow to verify the effects of the intervention in a prehabilitation context and analyze its effects on the post-surgery recovery of the participants in the follow-up assessment. More studies analyzing the effects of exercise on chemotherapy completion rates and tumor regression in BC are needed. The study results may represent less time to treatment exposure, quicker return to everyday life, fewer side effects, and better overall and disease-related quality of life.

**Clinical Trial Registration:**

clinicaltrials.gov, identifier NCT06429189.

## Introduction

### Background and rationale

Breast cancer (BC) is the most commonly diagnosed cancer type among women worldwide, showing high levels of incidence, prevalence, and cancer-related deaths (1). Chemotherapy (i.e., intravenous administration of a combination of cytotoxic drugs to delay cancer growth or, in some cases, eradicate the tumor and prevent cancer cell multiplication, invasion, and metastasizing) is a commonly used treatment for BC and neoadjuvant chemotherapy (NACT) (i.e., systemic treatment pre-surgery) is being incrementally used in women with early BC, according to international guidelines, to allow better cosmetic outcomes and reduce postoperative complications (e.g., lymphedema) (2, 3).

However, chemotherapy drugs are not selective to tumor cells or normal cells, and their side effects may hinder the functional performance (i.e., a person’s ability to perform daily activities involving physical effort with vigor and without excessive fatigue) of women with BC (2, 4, 5). Also, it is important to consider that, during the treatment phase, some severe conditions may occur (e.g., neutropenia) that often lead to dose reductions and delays (6).

Recent studies have identified common adverse effects, such as neutropenia and neuropathies, which can lead to dose reductions or delays in women with breast cancer. Additionally, these toxicities appear to be more frequent in women displaying sarcopenia (7, 8). Exercise interventions have shown promising beneficial effects on neutropenia and neuropathy symptoms (9, 10). Also, evidence has pointed out that exercise appears to be a safe and effective cancer co-therapy, improving functional performance and body composition in women with BC during treatments, which may, in turn, contribute to increased treatment efficacy (11–13).

Concerning disease prognosis and chemotherapy treatment efficacy, a relative dose intensity (RDI) (i.e., the ratio of dose intensity delivered relative to the standard dose intensity for chemotherapy regimen per unit of body surface area, per unit of time (weeks)) ≥85% is desirable (14, 15). Lower chemotherapy RDI is associated with a lower tumoral response rate and potential of cure, which ultimately leads to reduced overall survivorship and worse prognosis (16). Some evidence suggests that exercise may modulate treatment response through its effects on the tumor, making it a potential tool to enhance the chemotherapy completion rate (i.e., reception of the planned treatment doses) (15). Also, some recent evidence has suggested that exercise as a prehabilitation strategy during NACT may augment tumor regression in patients diagnosed with esophageal cancer (17). Considering these findings, it is plausible that the same may happen with women diagnosed with breast cancer undergoing NACT.

Despite the well-known benefits of exercise on women with BC, this population often fails to comply with the recommended physical activity guidelines for various reasons (e.g., fatigue, conflicting commitments, sickness, pain, and lack of self-discipline, enjoyment, or interest) (18).

So far, evidence has shown that exercise does not disrupt chemotherapy delivery but has failed to demonstrate that exercise may increase chemotherapy completion rates (15). For instance, Sanft et al. (19) conducted a home-based exercise and nutrition counseling intervention on 173 women with BC undergoing either neoadjuvant or adjuvant chemotherapy, reporting no intervention effects in RDI compared to a usual care group.

Home-based exercise interventions (i.e., exercise performed inside or in the immediate surroundings of one’s home that can be structured or unstructured accordingly with the specificity of the prescription for the participants and supervised, facilitated, or unsupervised, depending on the level of presence of the exercise professional during the training process (20)) have shown to be a convenient and flexible strategy with high adherence and have proven to be effective and beneficial in improving functional performance, raising activity levels, reducing symptoms, and improving overall quality of life in women with a BC diagnosis (21, 22).

Studies investigating the effects of exercise interventions on chemotherapy completion rates as a primary outcome are still needed to substantiate further findings (15). Also, there is a scarcity of studies investigating the effects of structured and supervised home-based exercise programs on chemotherapy completion rate, pathological complete response (i.e., the absence of all detectable cancer cells in tissue samples collected post-treatment), or tumor regression in women with BC undergoing NACT.

### Objectives

This paper describes the protocol of a 2-arm pragmatic randomized controlled trial, Home-Combo, which will target women with a BC diagnosis undergoing NACT. The Home-Combo study primarily aims to investigate the effects of a structured, supervised, online home-based group exercise intervention conducted during the neoadjuvant chemotherapy treatment period on the chemotherapy completion rates and tumor regression of women with BC. Secondly, this study intends to analyze the impact of this intervention on pathological complete response to treatment, functional performance, body composition, physical activity levels, and quality of life. A 3-month follow-up will be performed to investigate the short-term evolution of the outcomes post-surgery and active lifestyle sustainability post-intervention.

### Trial design

This study will be a 2-arm pragmatic and superiority single-blinded randomized controlled trial with an intervention and an active control group, with a 1:1 allocation ratio. It will comprise a structured, supervised, online home-based exercise group intervention via Zoom, starting 1–2 weeks after chemotherapy treatments begin and continuing until 3–4 weeks post-treatment. Additionally, a 3-month follow-up will be performed. This protocol followed the Spirit guidelines (23). This trial is registered in clinicaltrials.gov (number: NCT06429189).

## Methods

### Study setting

The Home-Combo study will take place in the Algarve, and the sample will be recruited by medical referral from various public and private hospitals and oncologic organizations across the region.

The Home-Combo intervention will be conducted online to ensure participants’ safety during the neoadjuvant chemotherapy treatment phase, as they may have compromised immunity. Also, this option was made to attenuate participants’ burden caused by commuting requirements (24). Due to the specific timings between the first contact and study enrolment, participants will be enrolled continuously over the course of one year. Recruitment will continue until the targeted number of participants per group is achieved or at the end of the timeline. A scheme of the study timeline is presented in **Table 1**.

**Table 1:** Spirit flow diagram. Participant Timeline for the Home-Combo Study.

HOME-COMBO Women STUDY PERIOD					
Timepoint	Enrolment	Allocation	Post-allocation		
	T_-1_	T_0_	T_1_	T_2_	T_3_
	Month 1	Month 1	Month 3	Month 6	Month 9
Enrolment					
Medical referral of potential participants	X				
Potential participants contact	X				
Pre-study initial meeting – confirm interest	X				
Eligibility screening	X				
Informed consent	X				
Baseline assessments	X				
Blinded randomization and allocation		X			
INTERVENTIONS					
Structured/supervised home-based combined exercise intervention			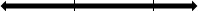		
Breathing and mobility exercises control group			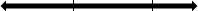		
ASSESSMENTS					
Primary outcomes (chemotherapy completion rate)		X	X	X	X
Tumor regression		X	X	X	X
Secondary outcomes (functional performance, quality of life, physical activity/sedentary behavior)		X	X	X	X
Chemotherapy complete response					X
Other outcomes (active lifestyle maintenance)					X

T_0_, 0-2 weeks post-neoadjuvant chemotherapy (intervention beginning)t; T_1_, mid-treatment; T_2_, 3-4 weeks post-chemotherapy (end of intervention); T_3_, 3-month follow-up.

### Eligibility criteria

Women aged ≥18 years with a BC stage I-III diagnosis, scheduled to receive NACT, and who have access to a device with internet will be considered eligible to participate in the study. Women who already had their second cycle of chemotherapy, with medical counterindication to perform the exercise intervention or physical assessments due to concomitant comorbidity, non-controlled health conditions or diseases, or psychological illness, currently enrolled in a structured exercise program, or women unable to complete the entire program (e.g., due to scheduled clinical or personal commitments) will be considered ineligible. Participants who get pregnant or have a worsening in their clinical condition that prevents them from continuing the exercise program safely, according to their primary physician’s evaluation, will be discontinued from the study’s program.

The exercise professionals performing the intervention must have an academic degree in exercise sciences an^17^d a specialization in exercise with cancer populations, or they must receive education sessions from an exercise professional specialized in training BC populations. Also, professionals who conduct the interventions will have to perform at least three simulated sessions among each other, supervised by a qualified peer, to guarantee consistency in the intervention application. Every training session in the intervention will follow a standardized exercise program. This approach ensures parsimony and consistency between all exercise professionals conducting the intervention.

### Interventions

#### Combined home-based exercise group

Participants will perform exercise throughout their chemotherapy treatments, starting within 1–2 weeks of the first treatment cycle and ending within 3–4 weeks post-treatment completion (25). The intervention program will include three unprecedented cycles, each consisting of two weekly sessions with distinct exercise plans: plan A on the first day and plan B on the second. These plans will rotate every two months, aligning with the average total duration of neoadjuvant therapy (approximately six months).

The exercise intervention will be led by qualified exercise professionals, comprising two weekly 60-minute online exercise group sessions with a 5-minute warm-up, a 30-minute resistance training, and a 20-minute cardiorespiratory exercise component, finishing with a 5-minute cooldown (17, 18, 26). All the sessions will be conducted live via Zoom and directly supervised by a qualified exercise professional. To maintain good vigilance and constant feedback from the professional to all participants, the groups will have no more than 8 to 10 participants. The intensity of the exercise program will be monitored using the Borgs Category-Ratio (CR) 10 scale of Rating of Perceived Exertion (RPE) (27, 28).

The warm-up will consist of three mobility exercises (e.g., doorway stretch, wall/floor slides, back scratch, piriformis stretch, cat-camel) and three activation movements (e.g., shoulder blade squeezes, pointers, shoulder external rotations), performed at light intensity (RPE 1-3) for 1 to 2 sets of 10 to 30 seconds (isometric exercises/stretches) or 10 to 15 repetitions (29, 30). For the resistance training, each participant from the intervention group will receive a kit with a suspension trainer, a set of elastic resistance tubes, and two sets of dumbbells of 3 and 5kg, respectively. The resistance training will focus on developing muscular endurance, consisting of 9 exercises, performed with either body weight or free weights. This component will comprise major movement patterns, such as pushing (e.g., chest presses, push-ups, shoulder presses, shoulder raises), pulling (e.g., double-arm and one-arm rows, leg curls), squatting (e.g., chair sit-to-stand), lunging, and lift (e.g., stiffed-leg deadlifts, glute bridges) and isolation movements (e.g., leg abductions, calf raises, toe raises, bicep curls), as well as core exercises (e.g., crunches, dead bugs, leg raises, crisscross). All exercises will be performed in 2–3 sets of 10–15 repetitions, resting 60 seconds between sets, performed at moderate to vigorous intensity (RPE 4-8) and in a controlled manner (2 seconds per movement phase) (25, 29, 30). The cardiorespiratory component will consist of continuous aerobic, low-impact dance exercises, moving large muscle groups to increase heart rate, involving marching, side steps, knee raises, leg curls, multi-directional arm movements, and dance and aerobic movements performed at moderate to vigorous intensity (RPE 4-8) (Winters-Stone et al., 2022; Grummt et al., 2024). Cooldown will comprise breathing exercises and active isolated stretching techniques for the upper and lower limbs performed at light intensity (RPE 1-3) (26, 30, 31). The Home-Combo exercise program details can be found in online **Supplementary Material 1**.

The planned exercise protocol will be adapted in order to consider exercise preferences, perceived barriers, and facilitators identified by women with BC, and suggestions from healthcare professionals working directly with oncology patients. This information will be collected before the intervention through a mixed-methods qualitative study that will include a survey answered by women who have or had a BC diagnosis and focus groups with healthcare professionals.

Before the beginning of the exercise program, participants will receive an education session on how to assemble the suspension trainer and elastic resistance tubes and how to use Borg’s CR-10 scale of RPE to monitor their effort during the training sessions. During the training sessions, participants in the intervention group will be instructed to stop exercising and report to the supervising professional whenever they feel excessively tired or experience dizziness, difficulty breathing, joint discomfort, or pain. The exercise professional will suggest initial loadings following a conservative approach and motivate the participants to increase loads when maximum repetitions are achieved, maintaining good technique and form, without excessive discomfort. Exercises will be adapted if women experience pain or discomfort due to a previous injury or treatment side effects. Also, loading will be adapted in cases where a central venous catheter is inserted to avoid malposition or displacement (32). Attendance to the sessions will be registered through the session’s recording. Additionally, women in this group will be encouraged to perform brisk walking at their preferred intensity and report in their activity logs (26, 33). The full program used in the intervention group can be found online in **Supplementary Material 2**.

#### Control group

During the intervention period, women randomized to the control group will receive one weekly 40-minute supervised session comprising light stretches and mobility exercises, breathing exercises, meditation, and relaxation exercises (26), throughout the entire neoadjuvant treatment phase (i.e, 8 sessions, covering the total average duration of this phase). Thus, the control group will act as an active comparator in this study. The light stretches and mobility exercises will be chosen based on traditional stretches, clinical Pilates movements, and soft yoga poses performed at an RPE 1-3. Breathing exercises will include basic, yogic, and meditative breathing techniques. Relaxation exercises will include progressive muscle relaxation techniques, visualization, and mindfulness exercises. Meditations cover various themes. Women in this group will be advised to maintain their normal routines and usual care outside the program sessions. Sessions will be recorded, and attendance will be registered. The sessions conducted with the control group can be found in online **Appendix 2**.

#### Criteria for discontinuing or modifying allocated interventions

In the initial pre-study meeting, participants will be informed that they may leave the study at any time. They will also be asked to refrain from continuing the intervention if a worsening clinical condition prevents them from exercising and performing the assessments safely. The exercise program will be tailored to the participants’ condition across the intervention. Participants’ adherence to the intervention and control group will be continuously monitored.

#### Strategies to improve adherence

Before implementing this study, a survey and focus-group-based qualitative study will be performed to adjust the exercise program according to the preferences, perceived barriers, and facilitators of women with a BC diagnosis, considering the environmental and cultural context where the intervention will take place, and healthcare professionals’ opinions regarding safety issues and potential barriers and facilitators that their patients may report. To attempt dropout minimization, we will have an active control group.

Women from the intervention group will be informed that if they remain through the entire study and participate in all the assessment moments, they will keep the equipment kit after the study is finished.

#### Concomitant care

Physiotherapy treatments prescribed by the participant’s primary physician and the physiotherapist will be allowed during the intervention. Participants will be asked not to engage in other exercise and physical activity programs or activities outside the program besides prescribed physiotherapy. Participants in the control group will be asked to maintain their regular routines outside the program’s activities.

#### Outcomes

Assessments will be performed at four time points: baseline (T_0_, 1–2 weeks post-chemotherapy start), mid-treatment (T_1_, planned mid-cycle), post-intervention (T_2_, 3–4 weeks post-chemotherapy finishing), and three months follow-up (T_3_). A non-interventionist research team member will control each participant’s assessment timings at each time point. The primary outcomes of this study will be the chemotherapy completion rates and tumor regression. Secondary outcomes will include the pathological complete response to treatment, functional performance, body composition, PA levels, sedentary behavior (SB) (i.e., awake behaviors with energy expenditure ≤1.5 METs while sitting or lying (34)), and quality of life. Demographical information (age, education level, marital status, socioeconomic status), PA history, and clinical history data (disease and treatment history, concomitant comorbidities, and other existing health problems besides BC) will also be collected.

### Sample size

Sample size calculations were performed in G*Power 3.1. program. Since no study was found that has specifically calculated the sample size focusing on chemotherapy completion rates as the primary outcome, calculations were performed to observe a medium effect size (d= 0.25) and statistical power of 80%, using a factorial analysis of variance (ANOVA) with repeated measures as the reference statistical test, giving an initial estimation of 86 participants (35, 36). From the research team’s experience with study dropouts in exercise trials with these samples, a 20% attrition rate was considered in the sample calculations, leading to a final estimation of 98 participants. Based on the findings of previous studies that analyzed the effects of pre-habilitative exercise on tumor regression in cancer patients undergoing NACT, the authors assume this sample size will be sufficient to allow meaningful analysis of the intervention on this outcome (17, 37).

### Recruitment

Recruitment will occur through medical referrals from primary physicians of several public and private hospitals and non-profit oncology associations in the Algarve region. Additionally, the project will be presented at BC-themed congresses and events, and flyers with information about the study will be made to assist in disseminating the project and the recruitment process. A research team member will then contact patients referred by the doctors to receive detailed information about the study. Optionally, patients can call the research team directly or contact them through email if they prefer. After confirming eligibility criteria and interest in participating in the study, patients will be asked to attend an initial session where more information will be given, and the informed consent will be signed. During that session, participants will be told they are not obliged to participate in the study and may decide to leave the project. Consent for data collection or sharing will also be obtained.

### Assignment of interventions

#### Allocation

##### Sequence generation

After signing the informed consent form, participants will be given an ID number, which they will use to identify the questionnaires, and assessors will use it to register their assessment results. A non-interventionist research team member will randomly assign the participants to either the intervention or control arm through an online program (https://www.graphpad.com/quickcalcs/randomize1/).

##### Allocation concealment mechanism

Due to the nature of the study, participant group concealment will be impossible. However, participants will only be randomized and informed about group assignment after the baseline assessment. Randomization and allocation will be performed by a non-interventionist research team member.

##### Implementation

After receiving all questionnaires, random allocation will be performed. The scheduling of the program’s activities will consider each participant’s timing of chemotherapy administration, guaranteeing no strenuous exercises for the women in the intervention group in the 48 hours following each cycle.

##### Blinding (masking)

The healthcare professionals responsible for prescribing and administering chemotherapy and registering all clinical information will be blinded to the participants’ groups.

### Data collection, management, and analysis

#### Data collection methods

Assessments will be conducted in standardized conditions, in a municipal sports center, in a calm and comfortable environment, in small groups, and performed by a qualified exercise professional and trained assistants. The assessments will take place in the morning, starting with body composition measurements, followed by a 15-minute pause so participants can eat (since they will be weighted while fasting), preceded by a 10-minute warm-up with general movements to mobilize big muscle groups and the physical tests, performed in the following order: shoulder angular measurements, strength, mobility, and cardiorespiratory endurance. If the number of participants is large, they will be divided into small groups to facilitate instruction and the conduction of the tests. After the field measurements, all questionnaires will be sent through e-mail and answered in Google Forms. Participants will be contacted 48 hours before the assessment to confirm their availability and presence. Summarized information regarding assessment time points and tools can be found in **Table 2**.

**Table 2:** Measurement details and time points.

	Assessment tool	Baseline T0	Mid-cycle T1	Post-intervention T2	3-month follow-up T4
Primary outcome					
Chemotherapy completion rates	Medical records	X	X	X	
Tumor regression	Medical records	X	X	X	X
Secondary outcomes					
Functional performance	6-MWT^1^ (aerobic endurance)	X	X	X	X
	Arm curl and sit-to-stand (upper and lower body strength)	X	X	X	X
	Dynamometer (hand grip strength)	X	X	X	X
	Timed up-and-go (mobility)	X	X	X	X
	Goniometry (shoulder flexion and abduction angular measures)	X	X	X	X
Body composition	Bioelectrical impedance	X	X	X	X
	Stadiometer (height)	X			
^4^PA levels	^5^IPAQ-SF	X	X	X	X
Quality of life	EORTC QLQ-C30^2^ EORTC QLQ- BR45^3^	X	X	X	X
Covariates					
Demographic information	General questionnaire	X			X
Clinical history	Medical records	X			X
PA history	General questionnaire	X			X

^1^6-MWT, 6-minute walk test; ^2^EORTC QLQ-C30, European Organization for Research and Treatment of Cancer Quality of Life Questionnaire; ^3^EORTC QLQ-BR45, EORTC QLQ specific breast cancer module; ^4^PA, Physical Activity; ^5^IPAQ-SF, International Physical Activity Questionnaire Short-Form.

#### Primary outcomes

##### Chemotherapy completion rates

Chemotherapy completion rates will be assessed at T_0_, T_1_, and T_2_. All data will be acquired from medical records, and information will include the date and dose of each drug administered, reasons for dose adjustments, and/or dose deals in the applicable cases (19). RDI will be expressed as a percentage of the planned chemotherapy dose intensity divided by the dose intensity prescribed using the following formulas (19): Planned total dose (mg)/[BSA (Body Surface area (m^2^)) x planned number of weeks on treatment]. The actual dose will be calculated using the formula (Sanft et al., 2023): Total dose delivered (mg/BSA x actual number of weeks on treatment). RDI will be calculated for each individual administered drug and posteriorly averaged across all participants’ chemotherapy drugs. Also, categorical completion outcomes will be created and documented, including (1) RDI <85%/≥85% (2), dose reductions (3), dose delays (4), duration of dose delays (5), combination of dose reductions, skips, termination ahead of the scheduled time, and/or delays (61), reasons for treatment dose reductions or delays (Sanft et al., 2023).

##### Tumor regression

Tumor regression information will be collected from medical records at T_1_, T_2_, and T_3_. The baseline value will be the originally measured and diagnosed tumor size, nodes affected, and metastasis detected. Information on tumor evolution will be collected, including tumor size comparisons, affected areas, and affected nodes. Tumor regression will be assessed using the residual cancer burden (RCB) scoring system, which will be used to analyze residual lesions after NACT based on breast tumors and regional lymph nodes (38). To calculate the RCB index values from the primary tumor bed area (mm x mm), overall cancer cellularity (%), percentage of cancer that is *in situ* disease (%), number of positive lymph nodes, and diameter of the largest nodal disease, will be inputted in a network calculator (https://www3.mdanderson.org/app/medcalc/index.cfm?pagename=jsconvert3, classified according to the cutoff values of 1.36 and 3.28, and categorized into four classes: RCB 0 (equal to pathological complete response), RCB I (minimal burden, >0, ≤ 1.36), RCB II (moderate burden, >1.36, ≤ 3.28), RCB III (extensive burden, > 3.28) (38, 39).

#### Secondary outcomes

##### Pathological complete response to treatment

The pathological complete response will be assessed at T4. It is measured by histopathological assessment according to the TMN (Tumor/Metastasis/Nodes) classification of the American Joint Committee on Cancer (ypT0/is ypN0) (40). The prefix “yp” indicates the staging is being performed after NACT, and ypT0/is and ypN0 indicates there’s no residual invasive tumor (T0) or only carcinoma *in situ* (Tis) at the primary site and no cancer is found in the lymph nodes (41). The pathology report deriving from the histopathological assessment will be obtained post-surgery, and the pathological complete response will be marked as positive if no residual invasive disease is noted in the pathologic specimen (ypT0N= or ypTisN0) (19).

Also, to classify the tumors post-neoadjuvant chemotherapy, the residual cancer burden (RCB) index will be used (42). The Residual Cancer Burden (RCB) is assessed and scored by breast cancer pathologists at the treatment centers. The RCB score is calculated as a continuous variable, with values ranging from 0 to over 3.28. A higher value indicates a larger amount of residual disease. The RCB index is categorized into different risk levels based on the extent of residual disease: RCB-0: no residual disease (pathologic complete response); RCB-I, minimal residual disease;

RCB-II, moderate residual disease; RCB-III, extensive residual disease.

##### Functional performance

Functional performance will be assessed at T_0_, T_1_, T_2_, and T_3_. The test battery will consider cardiorespiratory capacity, upper and lower body strength, grip strength, shoulder mobility, and overall mobility. Cardiorespiratory endurance will be assessed using the 6-minute walk test, a standardized field test performed indoors in a 30-meter corridor with two turning points marked every 3 meters along the course, that has been considered valid and reliable for evaluating cardiorespiratory capacity in cancer patients (43, 44). In this test, participants will be asked to walk the maximum distance possible in 6 minutes on a flat and hard surface, whereas the distance walked is the outcome (43, 44). The arm curl and sit-to-stand tests will be used to test the lower and upper body strength, respectively, since they are valid and reliable in these populations (45, 46). Handgrip strength will be measured using a dynamometer (Camry ISO 9001) since it has shown reasonable validity and reliability in measuring this variable among adults ≥50 years old and used in oncologic samples (47, 48). While performing the handgrip test for each hand, participants will be instructed to stand upright with feet at hip width and elbows completely stretched while applying the maximum grip continuously for more than 3 seconds, and 30 seconds of rest will be given between measurements (49). Handgrip strength will be considered to the nearest 0.1 kilograms, with each participant performing two attempts in each hand, and the best value will be considered (50). The timed up-and-go test will assess participants’ functional mobility. Participants will be instructed to rise from a chair, walk 2.44 meters, turn around, walk back to the chair, and sit down (45). Shoulder flexion and abduction angular measures will be performed on both sides using a validated and reliable goniometer to measure this variable (51). Upper limb mobility will be assessed using the back scratch test (45).

##### Body composition

Body composition will be measured through bioelectrical impedance (Tanita BC-601) at T_0_, T_1_, T_2_, and T_3_ at the beginning of each assessment (52). Data regarding weight, fat mass, lean mass, water percentage, bone mass, visceral fat, and phase angle will be collected on a scale. Participants will be asked to maintain their dietary patterns before the test and refrain from intense exercise the day before (52). The measurement will be performed under standardized conditions (i.e., in the morning, 8-hour fasting, bladder voiding before the assessment, controlled room temperature, and no exercise before the measurement) (53). The height measurement will be considered to the nearest 0.1 cm of the participants and performed using a balance-mounted stadiometer (SECA, Germany), with the participants standing and bare-footed (54). BMI (kg/m2) will be calculated from weight (kg) and height (m). Hip and waist measurements will be performed using a precision digital measuring tape to the nearest 0.1 centimeters. Hip-to-waist ratio will be calculated (waist measurement/hip measurement).

##### Physical activity and sedentary behavior

PA levels and SB will be assessed at T_0_, T_1_, T_2_, and T_3_ using the International Physical Activity Questionnaire Short-Form (IPAQ-SF) (55). The IPAQ-SF comprises nine items that measure the weekly time spent on all intensities of PA (i.e., light, moderate, and vigorous) and time spent sitting on weekdays and weekends. Total PA scores are calculated from the collected data and discriminated by intensity (55).

##### Quality of life

Quality of life will be assessed at T_0_, T_1_, T_2_, and T_3_, using the European Organization for Research and Treatment of Cancer Quality of Life Questionnaire (EORTC QLQ-C30). The specific module EORTC QLQ-BR45 will measure BC-related quality of life. Both tools demonstrated adequate validity and reliability, specifically for cancer patients (56, 57). The EORT QLQ-C30 questionnaire consists of 30 items, grouped into eight multi-item scales (i.e., functional: physical, role, emotional, cognitive, and social; symptom: fatigue, pain, and nausea), one global health status and quality of life subscale, and six single-item questions (dyspnea, insomnia, appetite loss, constipation, diarrhea, and financial difficulties) (56). The BC module QLQ-BR45 comprises five functional sub-scales (body image, future perspective, sexual functioning, sexual enjoyment, breast satisfaction) and seven symptoms sub-scales (arm symptoms, breast symptoms, endocrine therapy, skin mucositis, endocrine sexual symptoms, systemic therapy side effects, and upset hair loss), for a total of 45 items (the previous 23 items found in QLQ-BR23 plus 22 new items) (57, 58). High scores on the functional scales determine better functioning, and high scores on the symptoms scale and body image mean higher issues (56). Because the QLQ-BR45 algorithm has not yet been validated, the QLQ-BR23 algorithm will be used to score the questionnaire (59).

##### Covariates

###### Demographics, physical activity, and medical history

Participants’ demographic information (e.g., age, education, marital status, and economic status) and PA history will be collected through a general information questionnaire, and clinical history data will be retrieved from their medical records after they sign the informed consent form.

###### Plans to promote retention

A research team member will contact participants who fail to attend a session to ensure their welfare and motivate them to participate in the next session. All the participants from the intervention group will receive equipment to train at home and will be informed they can keep after the study is finished. Participants will receive positive feedback during the sessions, as positive feedback enhances feelings of competence, enjoyment, and interest in the activity (60). Additionally, participants will be encouraged to keep an activity diary where they may register all activities performed autonomously. After the intervention, participants will be contacted monthly to check their well-being and keep their interest and motivation in engaging in the follow-up assessment. Women in the intervention group will receive wellness sessions with light mobility, breathing and relaxation exercises.

### Data management

To guarantee the anonymity of the participants, the data collected from the medical records will be presented with the patient’s health system number.

All the data collected in this study will be kept confidential, computerized, and encrypted in a database without any elements that may allow identification of the participants. After giving written informed consent, an ID number will be provided to each participant. When the participant receives her ID number, all data inserted in the databases will not be directly linked to the participant’s identification. A dataset will be created for each assessment time point. All datasets will be maintained by the members responsible for the investigation on a secure server of CIDEFES-UL for ten years and will be used exclusively for research purposes. Datasets used for specific analyses or to develop sub-studies will contain only the necessary variables and the demographical indicators provided to the research team members upon request to the leading investigator. Also, this data will be available for other authors or studies upon request, aligning with open science best practices.

### Statistical methods

All data will be analyzed using IBM SPSS (version 29.0). The chemotherapy completion rates will be analyzed as a categorical variable (i.e., completers vs. non-completers, percentage of participants who had dose reductions/delays, percentage of participants who had dose reductions/delays due to health complications) using a Fisher’s exact test for this purpose, and as a continuous variable (i.e., actual RDI in the participants and specific percentage of dose reductions, in general, and per drug administrated, and average time of dose delays). Factorial ANOVAs with repeated measures will be used to analyze both primary and secondary outcomes, except pathological complete response to treatment that will be analyzed as a categorical variable through a Fisher’s exact test (61). Adjustments will be made for covariates, including, age, marital status, education level, concomitant treatments, BC stage, drugs used, and baseline physical activity levels, to control for confounding factors. Independent sample T-tests will be used to compare results between groups at each time point, considering chemotherapy completers versus non-completers (61). An intent-to-treat analysis will be conducted to ensure that all participants are included in the overall assessment. A per-protocol analysis will also be conducted without participants who failed to complete at least 50% of the training sessions. Normality plots and Kolmogorov-Smirnov tests will be performed to test the normality of outcome variables. If normality is not satisfied, non-parametric tests will be applied (e.g., Kruskal-Wallis). To manage type I errors during multiple testing, Bonferroni will be applied.

### Monitoring

#### Data monitoring

A data monitoring committee will not be required for this trial as the interventions pose minimal risk, and participants will be protected by insurance throughout the study.

#### Harms

All participants will have their adverse events monitored throughout the study, whether directly related to the intervention or not (if applicable). This monitoring will be done through self-reporting at the start of each session, registered for analysis and report purposes, or by their primary care physicians. Participants will be advised to contact the clinical team if they have difficulties. Also, during all intervention periods, participants will be covered by a personal accident insurance.

#### Auditing

Two authors will supervise all trial procedures and cross-check the interventionist actions and study processes. Additionally, an independent person external to the project will review the protocol.

## Discussion

The Home-Combo study aims to expand the knowledge regarding the effects of a supervised home-based combined exercise program for women undergoing neo-adjuvant for BC. The incidence of BC has been increasing steadily, along with the survival rates, over the years, particularly among women (1, 62). Home-based exercise training programs have already been proven safe for women with BC during the treatment phase (22, 63). Studies conducting structured and supervised home-based exercise programs in women with BC during the treatment phase are still scarce (22). Furthermore, since this study includes participants performing neo-adjuvant chemotherapy, it will be possible to verify the effects of the intervention in a prehabilitation (i.e., pre-operative) context and analyze its effects on the post-surgery recovery of the participants in the follow-up assessment. Also, according to Bland et al. (2019), there is a need for more studies that analyze the effects of exercise on chemotherapy completion rates. Also, analyzing the effects of exercise in tumor regression during the pre-operative stage is highly relevant, considering that some evidence has already suggested this effect for other types of cancer, and a larger tumor regression can ultimately mean a more conservative surgery and a significant reduction in postoperative complications (3, 17, 37). Therefore, this study may contribute significantly to robust knowledge regarding home-based exercise programs in this target population, and the effects of exercise on tumoral response to treatment in a pre-operative setting. Also, this study is expected to have immediate benefits to all women with BC enrolling in this study and a potential impact on improving interventions designed to promote higher adherence to exercise programs and active lifestyle behaviors.

Offering an online alternative and disseminating an easy-access exercise program that can be performed at home with safety and efficacy might be significant for contexts where public transportation is scarce or for people living in remote areas with little to no access to sports or exercise facilities may be relevant. If the hypotheses of the study are confirmed, or if results show at least that women with a BC diagnosis can stick to the planned chemotherapy sessions and doses, with little to no interruptions and no dose adjustments, these results will potentially represent less time to treatment exposure, quicker return to everyday life, fewer side effects, and better overall and disease-related quality of life. Also, if women performing exercise while on neoadjuvant chemotherapy experience a greater tumor regression and complete pathological response, medical professionals may recommend exercise practice from day one, with confidence and with a powerful argument that it may positively change the lives of BC patients. Exercise has proven to be safe during treatment, helping to manage many of the treatment side effects. If, on top of this information, it is shown also to improve treatment response, the findings of this study may be a large step further in confirming exercise as an oncologic co-therapy in BC. However, it is important to consider that since this study will focus on a specific population, generalizability of findings to other groups will not be possible.
