# Supplementary material for: Home-Combo: an online home-based exercise intervention for women with breast cancer undergoing neoadjuvant chemotherapy: study protocol for a 2-arm pragmatic randomized controlled trial
Source: Front Oncol. 2025 Dec 12;15:1682839. doi: 10.3389/fonc.2025.1682839 (PMC12740911; doi:10.3389/fonc.2025.1682839)
Supplement: Supplementary file 1 [file SupplementaryFile1.pdf]

EXERCISE PROGRAM: HOME-COMBO

CYCLE 1

Training plan A

Warm-up (5min)

1-2 sets per exercise

1- Doorway chest stretch (10-30 seconds)

2- Standing forward bend (10-30 seconds)

3- Wall/ floor slides (10-15 reps)

4- Foot series (Pilates) (10-15-reps)

5- Wall push-ups (10-15)

Resistance training (30 min)

2-3 sets, 10-15 repetitions, 60-second rest; execution tempo 2/0/2/0

1- Chest press (elastics)

2- Sit-to-stand/ squat (no equipment/ dumbbell)

3- Double arm back rows (elastics)

4- Glute bridge (no equipment/ dumbbell)

5- Upright rows (no equipment/ dumbbell)

6- Leg abductions (right/left)

7- Adapted crunches

Cardiorespiratory training (20-min)

2 rounds of 1 minute per exercise

1- March (mid-knee/high-knee)

2- Side step (with or without arm movement)

3- Leg curls (with or without arm movement)

4- Adapted jumping jacks

5- Rest

6- Reach down/ reach up

7-Step touch

8- V's (aerobic step)

9- Crossed steps

10- Rest

Cooldown (5 min)

2-minute breathing exercises

1- Diaphragmatic breathing (4/0/8/0)

3-minute active isolated stretch (5-10 reps per stretch, stretch hold 2 seconds per rep)

1- Shoulder adduction stretch

2- Back Scratch

3- Bend and reach (or seat and reach)

4- Calf stretch

Training plan B

Warm-up (5min)

1-2 sets per exercise

1- Lying butterfly chest stretch (10-30 seconds)

2- Floor angels (10-15 reps)

3- 90/90 hip switches

4- Pointer (10-15 reps)
5- Clams lv.1 (Pilates) (10-15 reps)

Resistance training (30 min)

2-3 sets, 10-15 repetitions, 60-second rest; execution tempo 2/0/2/0

1- Push-ups (Wall or suspension trainer)

2- Split squat (right/left) (no equipment/dumbbell)

3- Back rows (suspension trainer)

4- Shoulder flies (no equipment or dumbbells)

5- Lying leg curls (no equipment/elastic or dumbbells)

6- Bicep curls (no equipment/dumbells)

7- Calf raises (no equipment or dumbells)

Cardiorespiratory training (20-min)

2 rounds of 1 minute per exercise

1- Alternated leg repeaters (with or without arm movement)

2- Pendulum

3- Back steps with lunging

4- March (mid or high knees)

5- Rest; execution tempo

6- Side cross steps

7- Chassé

8- Forward and backward marches (5-10 steps in each direction)

9- Alt. knee raises (with or without arm movement)

10- Rest; execution tempo

Cooldown (5 min)

2-minute breathing exercises

1- Box breathing (4/4/4/4)

3-minute active isolated stretch (5-10 reps per stretch, stretch hold 2 seconds per rep)

1- Back Scratch

2- Neck stretches

3- Lying hamstring stretch (straight leg)

4- Hip twist stretch

CYCLE 2

Training plan A

Warm-up (5min)

1-2 sets per exercise

1- Shoulder circles (10-15 reps backward and forward)

2- Spine twist (10-15 reps)

3- Pilates arm openings lv. 1 in sitting (Pilates) (10-15 reps)

4- Cleopatra (Pilates) (10-15 reps)

5- Standing leg curls (10-15 reps)

Resistance training (30 min)

2-3 sets, 10-15 repetitions, 60-second rest; execution tempo 2/0/2/0

1- Chest press (Dumbbells)

2- Alt. Lunges (no equipment or dumbbells)

3- Back rows (Dumbbells)

4- Glute bridge (no equipment/ dumbbells)

5- Shoulder press (no equipment or dumbbell)

6- Alt. lateral lunges (no equipment or dumbbell)

7- Bicep curls (dumbbells)

8- Adapted crisscross

Cardiorespiratory training (20-min)

2 rounds of 1 minute per exercise

1- Forward cross steps (with cross-arm movements)

2- Alt. knee raise

3- Open hand jabs with foot movement

4- Walking series (Pilates)

5- Rest;

6- March (mid or high knees)

7- Adapted jumping jacks

8- Wall mountain climbers

9- Fast low knee marches

10- Rest;

Cooldown (5 min)

2-minute breathing exercises

1- Diaphragmatic breathing with glottis contraction (ujjayi breathing)

3-minute active isolated stretch (5-10 reps per stretch, stretch hold 2 seconds per rep)

1- Quads stretch (right leg)

2- Quads stretch (left leg)

3- Doorway stretch

4- Forward bend hamstring stretch

Training plan B

Warm-up (5min)

1-2 sets per exercise

1- All 4s spine twist (10-15 reps)

2- Cat-Camel (10-15 reps)

3- Hip circles in all 4s (10-15 reps)

4- Shoulder external rotation (elastic) (10-15 reps)

5- Pointers

Resistance training (30 min)

2-3 sets, 10-15 repetitions, 60-second rest; execution tempo 2/0/2/0

1- Alternated cross (diagonal) lunges

2- Chest flies (elastics)

3- Sit-stand/ squat (suspension trainer/dumbbell)

4- Back flies (elastics)

5- Leg abduction (right/left) (no equipment or elastics)

6- Skull crusher (no equipment or dumbbell)

7- Oblique crunches (right/left)

Cardiorespiratory training (20-min)

2 rounds of 1 minute per exercise

1- Squat heel raises (adaptation of squat jump)

2- Alt. back step with forward arm raises

3- High knee march

4- Step touch with trunk rotation and arm movement (like chest press arm movement)

5- Rest;

6- Low ceiling march (half-squat (±45º) position)

7- Adapted skaters

8- Adapted skiers

9- Front kicks

10- Rest;

Cooldown (5 min)

2-minute breathing exercises

1- Forced exhalation breathing

3-minute active isolated stretch (5-10 reps per stretch, stretch hold 2 seconds per rep)

1- Back Scratch (right arm on top)

2- Back scratch (left arm on top)

3- Seat and reach (right leg stretched)

4- Seat and reach (left leg stretched)

CYCLE 3

Training plan A

Warm-up (5min)

1-2 sets per exercise

1- Overhead reach (without and with D variation) (10-15 reps)

2- Pilates arm openings lv.1 (10-15 reps)
3- Pilates hip twist lv.1 (10-15 reps)

4- Wall push-ups (10-15 reps)

5- Pointer (10-15 reps)

Resistance training (30 min)

2-3 sets, 10-15 repetitions, 60-second rest; execution tempo 2/0/2/0

1- Push-ups (wall or suspension trainer)

2- Straight leg deadlift (single or double dumbbell)

3- Back rows (suspension trainer)

4- Glute bridge (no equipment or dumbbells)

5- Back flies (right/left) (elastics)

6- Bicep curls (elastics or dumbbells)

7- Deadbug

Cardiorespiratory training (20-min)

1 min exercise/ 1 min march

1- Step touch with arm movement

2- March

3- Adapted jumping jacks

4- March

5- Alternated knee raises

6- March

7- Feet open and close (with or without half-squat)

8- March

9- Alternated back steps with half lunge with forward arm movements

10- March

Cooldown (5 min)

2-minute breathing exercises

1- Alternated nostril breathing (4/0/8/0)

3-minute active isolated stretch (5-10 reps per stretch, stretch hold 2 seconds per rep)

1- Wall chest stretch

2- Seated adductors stretch

3- Sitting forward, bend stretch

4- Spine rotation stretch

Training plan B

Warm-up (5min)

1-2 sets per exercise

1- Doorway chest stretch (10-30 seconds)

2- Standing arm openings (10-15 reps per arm)

3- Good mornings (mobility) (10-15 reps)

4- Pointer (10-15 reps)

5- Glute bridge (10-15 reps)

Resistance training (30 min)

2-3 sets, 10-15 repetitions, 60-second rest; execution tempo 2/0/2/0

1- Chest press (dumbbells or elastics)

2- Sit-to-stand (no equipment or dumbbells)

3- Back rows (dumbbells or elastics)

4- Alternating Single leg deadlift (aided by suspension trainer or with dumbbells)

5- Shoulder press (dumbbells or elastics)

6- Sumo Squat (no equipment or dumbbells)

7- Tricep pushdown (elastics)

8- Leg raises

Cardiorespiratory training (20-min)

1 min exercise/ 1 min march

1- Adapted skiers

2- Mach

3- Adapted skaters

4- March

5- Adapted jumping jacks

6- March

7- Squat heel raises (adaptation from squat jumps)

8- March

9- Alternated knee raises

10- March

Cooldown (5 min)

2-minute breathing exercises

1-Breath hold (up to 30 seconds)

3-minute active isolated stretch (5-10 reps per stretch, stretch hold 2 seconds per rep)

1- Runner's stretch

2- Side bend stretch

3- Wall lats stretch

4- Calves Stretch

*Each cycle lasts for 2 months and then switches. When cycle 3 is finished, the program returns to cycle 1.
**Exercises are adjusted accordingly to the potential existence of a central sub-clavicular catheter, mobility limitations, and/or other co-existing comorbidities.
