# Supplementary material for: Home-Combo: an online home-based exercise intervention for women with breast cancer undergoing neoadjuvant chemotherapy: study protocol for a 2-arm pragmatic randomized controlled trial
Source: Front Oncol. 2025 Dec 12;15:1682839. doi: 10.3389/fonc.2025.1682839 (PMC12740911; doi:10.3389/fonc.2025.1682839)
Supplement: Supplementary file 2 [file SupplementaryFile2.pdf]

CONTROL GROUP PROGRAM: HOME-COMBO

Session 1

Light stretching and mobility = 12 minutes

Light stretches: 2-3 sets per exercise; hold each stretch for 10-15 seconds

Mobility exercises: 2-3 sets, 15-20 reps

1. Neck stretches
2. Back Scratch
3. Chest opener (wall)
4. Cobra pose (yoga)
5. Cat-camel
6. Arm openings lv.1 (Pilates)
7. Spine twist (Pilates)
8. Wall angels

Breathing exercises = 8 minutes

2-3 sets, 5-10 repetitions/cycles

1. Diaphragmatic breathing
2. Box breathing (4/4/4/4 sec)
3. Alternating nostril breathing (in 4 sec/ 8 sec)
4. Ujjayi breathing

Meditation = 10 minutes

Theme of meditation: inner wellness

Anchor: breathing

Option 2: Revitalizing cellular light – visualize a soft light penetrating the cells weakened by the treatment

Relaxation exercises = 10 minutes

Muscular tensing and relaxing exercise (each muscle tense up for 5-10 seconds)

Session 2

Light stretching and mobility = 12 minutes

Light stretches: 2-3 sets per exercise; hold each stretch for 10-15 seconds

Mobility exercises: 2-3 sets, 15-20 reps

1. Butterfly chest stretch
2. Standing forward bend
3. Sit-and-reach
4. Side bend
5. Open book
6. Double leg stretch lv.1 (Pilates)
7. Thread the needle
8. Windshield wipers (soft)

Breathing exercises = 8 minutes

2-3 sets, 5-10 repetitions

1. Diaphragmatic breathing
2. Forced exhalation breathing
3. Equal breathing (inhale 4/exhale 4)
4. Bhramari breathing

Meditation = 10 minutes

Theme: strength and resilience

Anchor: heart

Option 2: Gratitude – focus on a positive detail of your day and focus on amplifying that sensation of positivity and gratitude.

Relaxation exercises = 10 minutes

Countdown from 50 (inhale deeply and exhale completely on each count, without making a mistake; if a mistake is made, start from the beginning).

Session 3

Light stretching and mobility = 12 minutes

Light stretches: 2-3 sets per exercise; hold each stretch for 10-15 seconds

Mobility exercises: 2-3 sets, 15-20 reps

1. Viparita karani (lying with lifted legs pose)
2. Balasana (childs pose)
3. Baddha Konasana (bound angle pose)
4. Prayers stretch (on wall)
5. Toy solider (Pilates)
6. Mermaid in standing (Pilates)
7. Dum waiter (Pilates)
8. One leg circle (Pilates)

Breathing exercises = 8 minutes

2-3 sets, 5-10 repetitions

1. Diaphragmatic breathing (4 sec inhale/8 sec exhale)
2. Kapalabhati breathing
3. Alternated nostril breathing with breath holds (4 sec inhale, 8 sec hold, 4 sec exhale)
4. Mantra breathing (with significant word for the participant)

Meditation = 10 minutes

Sound guided meditation

Anchor: sound vibration

Option 2: Release resentment: forgiveness as freedom

Relaxation exercises = 10 minutes

Nature imagery exercise: guided beach visualization

Session 4

Light stretching and mobility = 12 minutes

Light stretches: 2-3 sets per exercise; hold each stretch for 10-15 seconds

Mobility exercises: 2-3 sets, 15-20 reps per side

1. Neck stretch
2. Overhead tricep stretch
3. Quads stretch
4. Calves stretch
5. Wall arm circles
6. Lying 90/90 shoulder external rotation
7. Over reach with "D" variation (Pilates)
8. Ankle rocks

Breathing exercises = 8 minutes

2-3 sets, 5-10 repetitions

1. Deep breathing
2. Box breathing (4-4-4-4)
3. Mental Anuloma Viloma (alternated nostril breathing)
4. 4-7-8 breathing (4 sec inhale; 7 sec hold, 8 sec exhale)

Meditation = 10 minutes

Mantra meditation: think of a word or sentence meaningful to you and repeat in your mind as you meditate

Achor: the word/sentence chosen

Option 2: Detachment – let go what does not serve any purpose

Relaxation exercises = 10 minutes

Body scan (guided mental scan of the body, relaxing each area, visualizing the tension melting going away)

Session 5

Light stretching and mobility = 12 minutes

Light stretches: 2-3 sets per exercise; hold each stretch for 10-15 seconds

Mobility exercises: 2-3 sets, 15-20 reps per side

1. Wall chest opener stretch
2. Shoulder blade stretch
3. Hamstrings stretch
4. Quads stretch
5. Upper body rolls (Pilates)
6. Side plie with stretch (Pilates)
7. Saw (Pilates)
8. Cat- camel

Breathing exercises = 8 minutes

2-3 sets, 5-10 repetitions

1. Alternating nostril diaphragmatic breathing
2. Alternating nostril kapalabhati breathing
3. Ujjayi breathing
4. Box breathing

Meditation = 10 minutes

Theme: mental clarity (reducing mind fluctuations)

Anchor: flow of breath in and out the nostrils

Option 2: Acceptance of the now, without resisting

"I don't need to like what's happening, but I can stay without running away"
Focus in acceptance of the present experience
Goal: reducing the suffering and cultivating presence

Relaxation exercises = 10 minutes

Yoga Nidra with visualization and countdown breathing

Session 6

Light stretching and mobility = 12 minutes

Light stretches: 2-3 sets per exercise; hold each stretch for 10-15 seconds

Mobility exercises: 2-3 sets, 15-20 reps per side

1. Gomukasana (Cow-faced pose)
2. Anantasana (reclining Vishnu couch pose)
3. Ardha matsyendrasana (half spine torsion pose)
4. Paschimottanasana (Sitting forward bend pose)
5. Floor angels
6. Overhead reach (Pilates)
7. Corkscrew (Pilates)
8. Double leg stretch (Pilates)

Breathing exercises = 8 minutes

2-3 sets, 5-10 repetitions

1. Pursed-lip breathing
2. Box breathing (4/4/4/4)
3. Bhramari breathing
4. Deep nasal breathing

Meditation = 10 minutes

Theme: colors (visualize a color in your mind. Choose a color that makes you feel good, in peace, or that gives you strength and resilience and reflect on the emotional impact of that color)

Anchor: chosen color

Option 2: Internal companion: I'm not alone – "Inside of me, there is a presence that never leaves me".

Focus: cultivating the sensation of internal support and emotional security

Goal: mitigate feelings of isolation and reinforce resilience

Relaxation exercises = 10 minutes

Visualization exercise: thoughts observation (try to become a spectator of your own thoughts, observing them without interacting, watching them come and go)

Session 7

Light stretching and mobility = 12 minutes

Light stretches: 2-3 sets per exercise; hold each stretch for 10-15 seconds

Mobility exercises: 2-3 sets, 15-20 reps per side

1. Butterfly chest stretch
2. Prayers stretch
3. Ushtrasana (camel pose)
4. Parsvottanasa (split leg forward bend pose)
5. Overhead reach w/ arm circles (Pilates)
6. Upperback warm-up (Pilates)
7. Upper body reaches (Pilates)
8. Roll Down (Pilates)

Breathing exercises = 8 minutes

2-3 sets, 5-10 repetitions

1. Diaphragmatic breathing
2. Alternated nostril breathing
3. Forced exhalation breathing
4. Zen breath counting (count each breath up to 10)

Meditation = 10 minutes

Theme: gratitude (think of things you are grateful for, and say, "I am grateful for…")

Anchor: gratitude objects

Option 2: Pain as a messenger, not as an enemy – I hear pain without fear. She shows me where to care.
Focus: conscious relation with physical or emotional pain

Goal: reduce reactivity, enlarge tolerance and understanding

Relaxation exercises = 10 minutes

Sound-guided relaxation (start by focusing on the external sounds and shift to your interior sounds)

Session 8

Light stretching and mobility = 12 minutes

Light stretches: 2-3 sets per exercise; hold each stretch for 10-15 seconds

Mobility exercises: 2-3 sets, 15-20 reps per side

1. Uttanasana (forward bend pose)
2. Trikonasana (triangle pose)
3. Ardha Matsyendrasana (half lord of the fishes pose)
4. Gomukasana (Cow-faced pose)
5. Floor angels
6. Arm openings (Pilates)
7. Thread the needle
8. Mermaid in sitting (Pilates)

Breathing exercises = 8 minutes

2-3 sets, 5-10 repetitions

1. Deep breathing

2.Ujjayi breathing

3. Kapalabhati breathing

4. Alternated nostril breathing with breath hold (4/12/8)

Meditation = 10 minutes

Theme: emotional healing (visualize a healing light involved)

Anchor: ball of light

Option: Flame of hope visualization, that grows at each breath in

Relaxation exercises = 10 minutes

Visualization exercise: think about the happiest moment in your life and try to visualize as many details as possible and associated feelings.
